# Low yield of unselected testing in patients with acutely abnormal liver function tests

**DOI:** 10.1177/2054270415611309

**Published:** 2015-12-02

**Authors:** Andrew Chadwick, Michael Marks

**Affiliations:** 1 St Thomas’ NHS Foundation Trust, London, SE1 7EH, UK; 2 Clinical Research Department, Faculty of Infectious and Tropical Diseases, London School of Hygiene & Tropical Medicine, London, WC1E 7HT, UK

**Keywords:** Clinical diagnostic tests

## Abstract

**Objectives:**

To audit the diagnostic yield and cost implications of the use of a ‘liver screen’ for inpatients with abnormal liver function tests.

**Design:**

We performed a retrospective audit of inpatients with abnormal liver function tests. We analysed all investigations ordered including biochemistry, immunology, virology and radiology. The final diagnosis was ascertained in each case, and the diagnostic yield and cost per positive diagnosis for each investigation were calculated.

**Setting:**

St Thomas’ NHS Trust.

**Participants:**

All inpatients investigated for abnormal liver function tests over a 12-month period.

**Main outcome measures:**

We calculated the percentage of courses due to each diagnosis, the yield of each investigation and the cost per positive diagnosis for each investigation.

**Results:**

A total of 308 patients were included, and a final diagnosis was made in 224 patients (73%) on the basis of both clinical data and investigations. There was considerable heterogeneity in the tests included in an acute liver screen. History and ultrasound yielded the most diagnoses (40% and 30%, respectively). The yield of autoimmune and metabolic screens was minimal.

**Conclusions:**

Our results demonstrate the low yield of unselected testing in patients with abnormal liver function tests. A thorough history, ultrasound and testing for blood-borne viruses are the cornerstones of diagnosis. Specialist input should be sought before further testing. Prospective studies to evaluate the yield and cost-effectiveness of different testing strategies are needed.

## Introduction

Liver disease is increasing and now accounts for 1.5% of deaths in the United Kingdom.^1^ As a result, the assessment of patients with both incidental and persistently abnormal liver function tests (LFTs) is an increasingly common clinical problem encountered by the acute physician. The use of a ‘liver-screen’ to test not only for viral causes of liver disease but also for metabolic and inherited conditions is common clinical practice,^2–4^ although there are limited data to support such an approach in the inpatient settings.

Studies in the community suggest that the yield of unselected testing is low. In studies of patients with incidental derangement of their LFTs, the yield of a ‘liver screen’ was between 3 and 10%.^5,6^ In contrast, a cause can be identified in over 75% of patients with persistently elevated LFTs.^7–9^ This suggests that biochemical liver screens can be safely delayed until a persistent elevation of LFTs is demonstrated. The only study of acutely jaundiced patients showed imaging and clinical course to be the two most important factors in making a diagnosis.^10^

While individual elements of a liver screen are relatively low cost, unselected testing may result in substantial costs at a national level,^11^ both from direct costs associated to testing and secondly in indirect costs due to prolonged inpatient stay, without a significant improvement in diagnostic yield.

The aim of this audit was to evaluate the diagnostic yield of investigations ordered as part of routine clinical care for inpatients investigated for abnormal LFTs at a large acute hospital.

## Methods

### Audit population

The hospital pathology records of every patient seen between 1 January 2011 and 31 December 2011 were reviewed. Requests for α-1-antitrypsin, caeruloplasmin and liver auto-antibiodies were used to identify patients undergoing an unselected liver screen. Patients were excluded if they were being investigated as an outpatient or aged under 18.

### Data collection

The electronic records system were used to obtain demographic data, and the results of the following tests for every patient: ultrasound liver, serology for Hepatitis A, B, C, D, E, HIV, CMV and EBV, liver auto-antibodies, caeruloplasmin, alpha-1-anti-trypsin, ferritin, ANA, immunoglobulins, LFTs and full blood count. Ultrasound reports were reviewed, and patients were categorised as having evidence of steatohepatitis, cirrhosis, biliary dilatation, gallstones, gallbladder wall thickening, ascites, portal hypertension and mass lesions. The cost of investigations was provided by the hospital laboratory department. This was used to calculate the cost per positive diagnosis of each investigation.

### Diagnoses

Electronic records, clinic letters and discharge letters were used to ascertain the clinical diagnosis for each patient, and where a clinical diagnosis was not given the clinical details and test results were reviewed by one author (MM) who assigned the patients to a diagnostic category.

### Ethical approval

This was a retrospective case note review of routine clinical data meeting the NHS definition of an audit^12^ and formal institutional review board approval was therefore not required.

## Results

A total of 308 had an inpatient request for at least one of liver auto-antibodies, caeruloplasmim, α-1-antitrypsin in 2011. The majority were male (n = 200, 65%) with a median age of 51.5 years (IQR 41–68). Median peak ALT, ALP and Bilirubin was 76 IU/L (IQR 33–294 IU/L, normal range 3–35 IU/L), 171 IU/L (IQR 89–299 IU/L, normal range 30 to 120 IU/L) and 23 mmol/L (IQR 9–70 mmol/L, normal range 3–17 mmol/L), respectively. On review of clinical records, no patient had a family history of Wilson’s disease or α-1-antitrypsin deficiency.

### Testing

The frequency with which elements of the Liver Screen were sent is shown in Table 1. No investigation was organised in greater than 90% of patients. Testing for all three common hepatitis viruses (A,B,C) was carried out in 157 patients (51%). The combination of an ultrasound and testing for viral hepatitis was carried out in 110 patients (36%). Despite national guidelines,^13^ an HIV test was only sent in 36% of patients who had testing sent for either Hepatitis B or C. Changes consistent with steatohepatitis was the commonest finding on ultrasound (n = 78, 41% of patients undergoing ultrasound, Table 2).

**Table 1.** Tests requested in investigation of abnormal liver function tests.

Test	Number (%) test performed	Number (%) diagnosis reached^a^	Cost per diagnosis
History	N/A	90 (40)	N/A
Ultrasound	189 (69)	67 (30)	£158
Hepatitis A	168 (55)	6 (3)	£857
Hepatitis B	238 (77)	9 (4)	£1044
Hepatitis C	233 (76)	15 (7)	£265
Autoimmune (all)	269 (87)	3 (1)	£2796
Metabolic tests (any)	145 (47)	0 (0)	^b^

a This shows the percentage of diagnosis reached due predominantly to the test in cases where a diagnosis was reached; n = 224

b No diagnoses were made via metabolic testing therefore a cost per diagnosis can not be calculated. α-1-antitrypsin, Caeruloplasmin and Ferritin as screening tests costs £18.42 per patient.

**Table 2.** Findings on ultrasound.

Finding	Frequency, %
Steatohepatitis	41
Splenomegaly	20
Ascites	20
Hepatomegaly	16
Gallbladder/biliary tract disease	16
Cirrhosis	13
Liver mass	5
Pancreatic abnormality	4

Note: 189 Patients underwent ultrasound.

### Diagnosis

**Figure 1.**
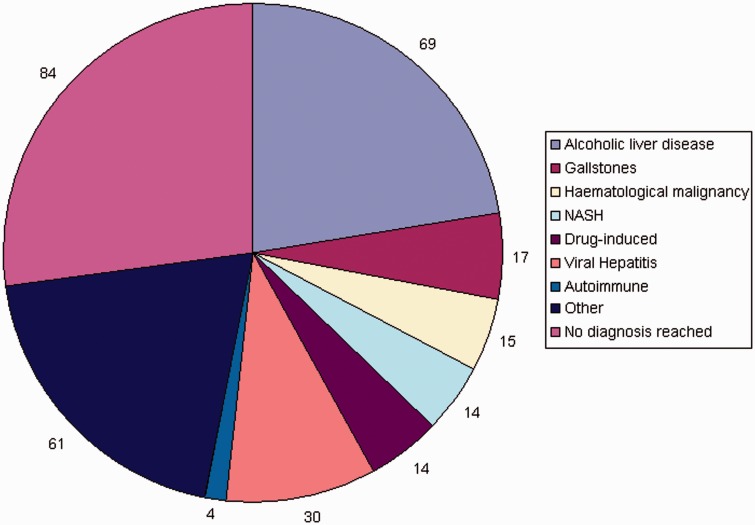
Diagnosis for abnormal liver function tests. Alcoholic liver disease and biliary disease were the major causes of abnormal liver function tests. The yield of autoimmune and metabolic testing was minimal.

No definitive diagnosis was made in 27% of patients despite investigation. Alcohol-related liver disease (22%), malignancies (11%), viral hepatitis (10%) and gallstone disease (6%) were the commonest identified causes of abnormal LFTs (Figure 1).

### Yield and cost of diagnostic testing

Ultrasound and viral serology were the tests with the highest diagnostic yield (Table 1). Measurement of caeruloplasmin, α-1-antitrypsin or ferritin did not contribute to the diagnosis in any cases. The cost to yield ratio varied from £158 per positive test with ultrasound to £2,976 per positive test with liver auto-antibodies (Table 1).

## Discussion

In this audit of investigations ordered as part of the routine clinical investigations of over 300 inpatients with abnormal LFTs, alcoholic liver disease was the commonest diagnosis made. Despite extensive investigation, no diagnosis was made in over a quarter of patients. History, ultrasound and testing for viral causes were the most useful elements of the diagnostic pathway. In this audit, there was no diagnostic value in measuring caeruloplasmin, α-1-antitrypsin or ferritin. The direct cost of these tests was substantial. Although our audit does not directly address the question of delayed discharges costs, it is plausible that additional testing may also contribute to indirect costs by prolonging inpatient stay.

**Figure 2.**
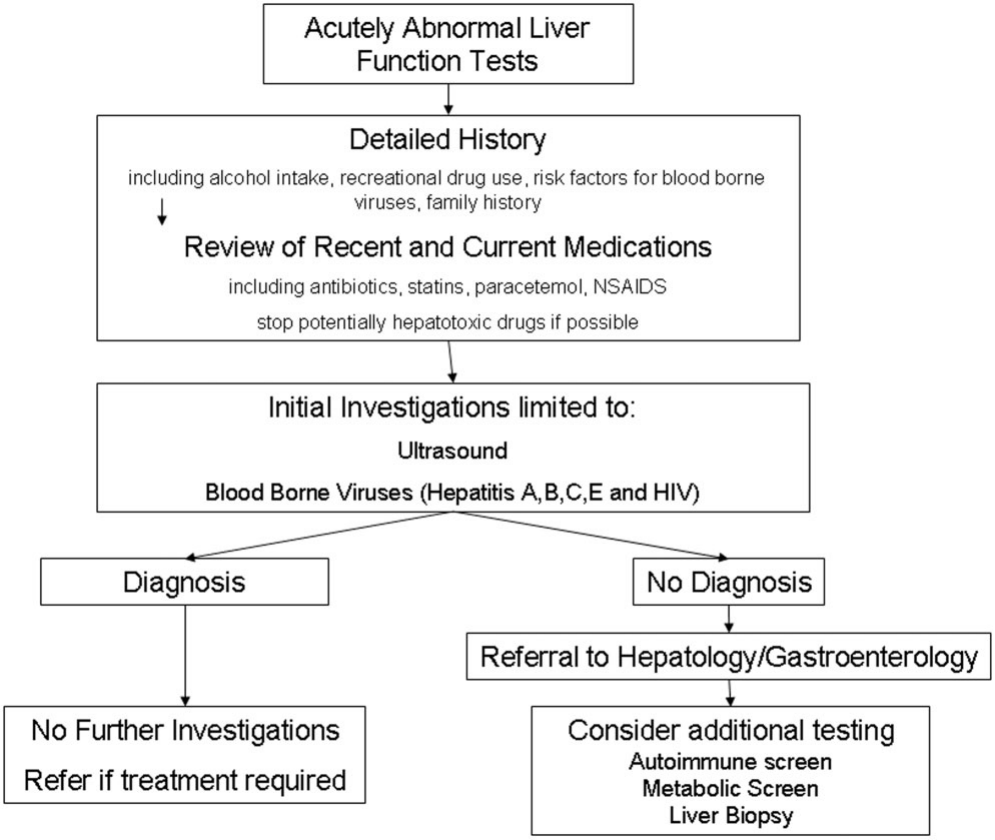
Approach to investigating abnormal liver function tests in inpatients. A detailed history and review of medications are key to establishing the underlying diagnosis. Initial diagnostic testing should be limited to imaging and blood-borne virus testing.

The second major finding is the heterogeneity of the tests ordered. Only 36% of patients underwent both an ultrasound and testing for hepatitis A, B and C. Furthermore, despite national guidelines for the routine testing of HIV, this was undertaken in only one-third of patients. These findings highlight the lack of consensus on the appropriate pathway for investigating inpatients with abnormal LFTs. We propose that a standardised approach be taken to investigating these patients (Figure 2). Such an approach would limit unnecessary testing and ensure that patients undiagnosed after initial investigation are referred to appropriate specialists for more detailed investigations such as caeruloplasmin where appropriate.

As this was a retrospective audit of clinical practice, the major limitation is that data are retrospective and partially incomplete. Patients were not systemically investigated, and we cannot be certain that a final diagnosis was not missed in a number of patients. In particular, it is likely that drug-induced liver function abnormalities accounted for a higher proportion of cases. Because investigations were not ordered systematically, we did not perform formal statistical comparisons of the diagnostic yield of each test, although the descriptive statistics clearly highlight history, ultrasound and blood-borne virus testing as the most useful diagnostic tools. As the aim of the audit was to assess the yield of unselected testing, only patients undergoing an unselected liver screen were included, rather than all patients with bilirubineamia or transaminitis, which may have introduced a selection bias. Some patients who underwent more targeted testing, in particular for viral hepatitis, may have been excluded. Such a bias would be likely to strengthen our core results in showing that targeted with history, ultrasound and virology yield the vast majority of diagnoses, while further decreasing the relative yield of biochemical and metabolic testing. While diagnoses were assigned on the basis of combined clinical and diagnostic data, ultrasound findings such as steatohepatitis can be non-specific. It is possible that some cases classified as alcoholic hepatitis on the basis of an appropriate clinical history and ultrasound findings may in fact have been due to other undiagnosed causes. Finally, this audit only included inpatients with abnormal LFTs. As such the findings may not be generalisable to primary care or outpatient settings where a different diagnostic pathway may be appropriate.

Despite these limitations, this audit includes a large number of both surgical and medical inpatients with abnormal LFTs and is likely to reflect everyday clinical practice. The findings of our audit are in agreement with a previous study in Denmark,^10^ which showed history and imaging to be most useful when investigating hyperbilirubinaemia. The percentage of patients in whom no diagnosis was made is also similar to previous studies. Clinical evaluation and directed testing diagnose nearly all patients with an identifiable cause for their abnormal LFTs. Our findings suggest that the routine use of unselected testing in patients with abnormal LFTs is both clinically unjustified and is likely to result in significant unnecessary expenditure on both direct and indirect costs. Approaches to decrease unselected testing such as informing clinicians of the cost of tests^14^ should be considered.

In conclusion, our audit demonstrates there is a considerable heterogeneity in the evaluation of inpatients with abnormal LFTs and that a number of commonly requested tests have minimal yield in the routine inpatient setting but result in substantial expenditure. Our audit highlights the need for a prospective evaluation of the yield and cost-effectiveness of different testing strategies to enable the development of a standardised approach to the investigation of inpatients with abnormal LFTs. Such an approach would be likely to improve both clinical and financial outcomes.

## Declarations

### Guarantor

MM
